# Systematic review and meta-analysis of maintenance of physical activity behaviour change in cancer survivors

**DOI:** 10.1186/s12966-019-0787-4

**Published:** 2019-04-27

**Authors:** Chloe Grimmett, Teresa Corbett, Jennifer Brunet, Jonathan Shepherd, Bernardine M. Pinto, Carl R. May, Claire Foster

**Affiliations:** 1 School of Health Sciences, University of Southampton, Southampton, UK; 2 Southampton Health Technology Assessments Centre, University of Southampton, Southampton, UK; 3 Faculty of Health Sciences, University of Ottawa, Ottawa, ON Canada; 4 College of Nursing, University of South Carolina, Columbia, USA; 5 Faculty of Public Health and Policy, London School of Hygiene and Tropical Medicine, London, UK

**Keywords:** Systematic review, Meta-analysis, Physical activity, Exercise, Cancer, Behaviour change, Behaviour change techniques, Maintenance, Intervention

## Abstract

**Background:**

Physical activity can improve health and wellbeing after cancer and may reduce cancer recurrence and mortality. To achieve such long-term benefits cancer survivors must be habitually active. This review evaluates the effectiveness of interventions in supporting maintenance of physical activity behaviour change among adults diagnosed with cancer and explores which intervention components and contextual features are associated with effectiveness.

**Methods:**

Relevant randomised controlled trials (RCTs) were identified by a search of Ovid Medline, Ovid Embase and PsychINFO. Trials including adults diagnosed with cancer, assessed an intervention targeting physical activity and reported physical activity behaviour at baseline and ≥ 3 months post-intervention were included. The behaviour change technique (BCT) taxonomy was used to identify intervention components and the Template for Intervention Description and Replication to capture contextual features. Random effect meta-analysis explored between and within group differences in physical activity behaviour. Standardised mean differences (SMD) describe effect size.

**Results:**

Twenty seven RCTs were included, 19 were pooled in meta-analyses. Interventions were effective at changing long-term behaviour; SMD in moderate to vigorous physical activity (MVPA) between groups 0.25; 95% CI = 0.16–0.35. Within-group pre-post intervention analysis yielded a mean increase of 27.48 (95% CI = 11.48-43.49) mins/wk. of MVPA in control groups and 65.30 (95% CI = 45.59–85.01) mins/wk. of MVPA in intervention groups. Ineffective interventions tended to include older populations with existing physical limitations, had fewer contacts with participants, were less likely to include a supervised element or the BCTs of ‘action planning’, ‘graded tasks’ and ‘social support (unspecified)’. Included studies were biased towards inclusion of younger, female, well-educated and white populations who were already engaging in some physical activity.

**Conclusions:**

Existing interventions are effective in achieving modest increases in physical activity at least 3 months post-intervention completion. Small improvements were also evident in control groups suggesting low-intensity interventions may be sufficient in promoting small changes in behaviour that last beyond intervention completion. However, study samples are not representative of typical cancer populations. Interventions should consider a stepped-care approach, providing more intensive support for older people with physical limitations and others less likely to engage in these interventions.

**Electronic supplementary material:**

The online version of this article (10.1186/s12966-019-0787-4) contains supplementary material, which is available to authorized users.

## Background

The incidence of cancer is increasing worldwide with an estimated 14.1 million diagnoses in 2012 and projections of 23.6 million new cases each year by 2030 [1]. With advances in early diagnosis and treatments survival rates are increasing. Thirty-two million people globally were alive at least 5 years following a diagnosis in 2012 [2]. Such advances are applauded, but the disease and its treatment can have long-term impact on a person’s physical and psychological health, including declines in physical function, cancer-related fatigue and poor quality of life [3].

There is good evidence that regular physical activity can improve many negative consequences reported by adults diagnosed with cancer [4, 5]. It is also well established that regular physical activity reduces the risk of developing comorbid disease, and delays decline in physical and mental functioning associated with aging [6]. Furthermore, accumulating observational evidence suggests regular physical activity may reduce cancer recurrence and mortality and prolong disease-free survival [7–10].

Consequently, adult cancer survivors have much to gain from being regularly active and it is recommended that they meet physical activity guidelines, that is to avoid inactivity and participate in at least 150 min of moderate intensity physical activity (or equivalent of vigorous activity) and two sessions of resistance exercise each week [11, 12]. Perhaps unsurprisingly, few cancer survivors meet these recommendations [13, 14] and they are less active than those without a history of the disease [15, 16].

Researchers in behavioural science are endeavouring to design interventions that promote regular physical activity among this population. Empirical evidence from a growing number of randomised controlled trials (RCT) suggests successful increases in physical activity on completion of interventions. In a meta-analysis of 12 interventions based on social cognitive theory, Stacey et al. [17] report a significant intervention effect for physical activity (SMD = 0.33; *p* < 0.01). Similarly, a systematic review and meta-analysis of 14 interventions designed to promote physical activity behaviour change in breast cancer survivors concluded that many interventions were effective in producing short-term improvements in physical activity [18].

However, to achieve long-term health benefits, behaviour change must be sustained and we know much less about effectiveness of interventions for promoting long-term change. For example, in a review of the maintenance of outcomes following physical activity and/or dietary interventions in breast cancer survivors only 10 (16%) of the 63 trials identified assessed outcomes at least 3 months after the intervention had ended. The majority of outcomes were physical or psychosocial variables, such as functional status, cancer-related fatigue and quality of life rather than measures of physical activity [19]. Jankowski et al. [20] synthesised the evidence of maintenance of outcomes, albeit not limited to physical activity behaviour, following physical activity interventions among cancer survivors in the hope of informing translation of research into practice within community settings. Only 12 RCTs reporting maintenance of intervention outcomes were identified, four included data on minutes of physical activity with two achieving significant improvements in behaviour.

Additionally, little is known about intervention components that might facilitate long-term behaviour change. Behaviour change interventions are frequently complex, consisting of numerous interacting components that are often poorly described [21]. This makes synthesising the evidence for effectiveness to inform future interventions challenging. In order to advance behavioural medicine by promoting precise and consistent reporting of complex interventions, Michie and colleagues [22] developed The Behaviour Change Technique Taxonomy (version 1). Based on expert consensus, this hierarchical classification system has been used in numerous systematic reviews to reliably identify behaviour change techniques associated with the most successful interventions (e.g. [23–25]. Coding the functions of existing interventions and the behaviour change techniques employed, and comparing these components across effective and ineffective interventions may help to identify the successful ‘active ingredients’ of interventions [26].

In addition to the contribution of Michie et al’s [22] theory-based BCT taxonomy, guidelines have been developed to improve reporting of interventions. The Template for Intervention Description and Replication (TIDieR) checklist highlights numerous contextual factors of intervention delivery which may impact intervention efficacy that the BCT taxonomy does not capture. Examples include mode of intervention delivery, such as home-based programs verses on-site supervised activity sessions, the intensity and duration of the intervention, and fidelity assessment [27]. Synthesis of both *intervention components* (through coding of BCTs) and *intervention context* (through the TIDieR checklist) and their association with longer-term physical activity behaviour could help inform development of future programmes.

When attempting to understand the optimal methods to support physical behaviour change, examination of RCTs has limitations. By only evaluating an intervention as ‘successful’ if there are statistically significant differences between intervention and control/comparison groups at follow-up we may ignore important changes in the control groups. It is well established that ‘contamination’ by control participants is common in trials of physical activity and dietary behaviour change [28]. Those who agree to participate are often highly motivated to make positive change, and thus may modify their behaviour irrespective of group allocation. This can lead to type II error, that is, an intervention being perceived as ineffective even when large increases in behaviour are achieved in the intervention group. This issue is then magnified by pooling such data in meta-analysis of RCTs. Furthermore, by relying on between group differences as a marker of success and then exploring intervention components and sample characteristics in order to explain the superiority of these interventions, we risk reaching erroneous conclusions as to what components might contribute to the most effective interventions. Trials of behaviour change often include ‘attention control’ designs and/or provide readily available printed materials promoting engagement in regular physical activity. By quantifying change in long-term behaviour in control groups, we get a sense of the amount of behaviour change elicited by these processes and by the act of consenting to a trial of physical activity in the context of cancer and recovery.

In this paper we describe the first reported systematic review and meta-analysis of RCTs in adults affected by any type of cancer, evaluating the efficacy of interventions on maintenance of physical activity behaviour change. Using the BCT taxonomy (v1) and TIDieR checklist, we attempt to identify both intervention components and contextual features that are associated with successful, post-intervention behaviour change to inform future intervention development. Meta-analysis of long-term change in physical activity behaviour in control groups is also presented and discussed.

## Methods

Guidance from the Centre for Reviews and Dissemination [29] and the Preferred Reporting Items for Systematic Reviews and Meta-analysis (PRISMA) [30] informed the methods for conducting and reporting this review. This review was registered with PROSPERO; CRD42017068924.

### Literature searching

**Fig. 1:**
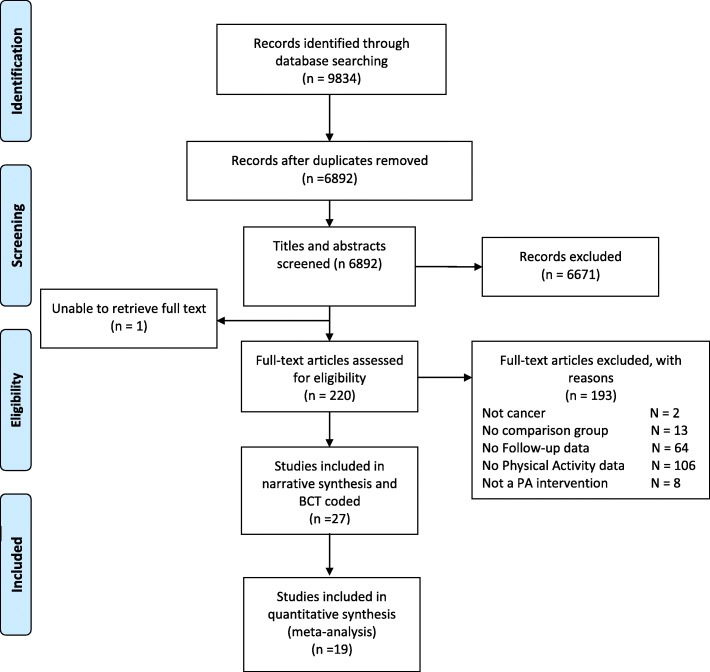
PRISMA flow

The following databases were systematically searched from inception to August 2018: Ovid Medline, Epub ahead of print, In Process & Other non–indexed citations, Ovid Embase and Ebsco PsycINFO. Conference proceedings were searched from 2015 to August 2018 via the Web of Science platform Science Citation Index & Social Science Citation Index & Conference Proceedings Citation Index. The strategy is a balanced combination of index and free text synonym terms, representing *cancer survivor*, *exercise, physical activity*, and *lifestyle interventions*. A clinical trial search filter was applied to the strategy to identify randomized or controlled trials or validation or evaluation studies. The search was limited to studies published in English. Reference lists of all included articles were hand searched for other relevant papers. The search results were screened on title and abstract against the inclusion criteria by two researchers (CG and TC). Full texts were obtained for publications identified to be potentially relevant and were screened independently by two reviewers (CG and TC); disagreements were discussed until a consensus was reached. Search hit returns can be referred to in the PRISMA chart (Fig. 1.). An example search strategy (Medline) is listed in Additional file 1.

### Study selection

The eligibility criteria for study inclusion were; 1) trials including adults (≥ 18 years) with a diagnosis of cancer, 2) trials that assessed an intervention that targeted aerobic physical activity (interventions limited to exercises for specific areas of the body, such as arm exercises for lymphoedema or pelvic floor exercises were excluded), 3) participants were randomised to an intervention and a control/comparison group, 4) reported outcomes data on physical activity behaviour (this could be expressed as an estimate of total energy expenditure (e.g. calories per kilogram per week (kcal/kg/week), METs per week (METs/wk) or minutes per week (mins/wk) of moderate to vigorous physical activity (MVPA) at the end of intervention measured directly or indirectly, and at least 3 months post-intervention completion with no intervention contact between the end of intervention and post-intervention follow-up. With no clear consensus on the definition of ‘maintenance’ of a behavioural outcome, the criterion of at least 3 months was selected as per previous systematic reviews of physical activity among cancer populations [31, 32]. The original protocol specified inclusion of pre-post studies however this was modified to include RCTs only to ensure optimal methodological rigour.

### Data extraction and risk of bias assessment

A data extraction form capturing study details and physical activity behaviour data was developed and iteratively refined by CG to ensure comprehensive data capture. One author (CG) extracted data on author, country of study, study design, sample size, population studied (including cancer type, age, comorbidities, ethnicity and level of education), intervention type (i.e. physical activity only or lifestyle intervention that was not restricted to physical activity), study duration, attrition rate, physical activity outcome measure and physical activity data. Features of the intervention were extracted based on the TIDieR checklist for reporting of interventions. Theoretical basis was also captured. The BCT taxonomy (v1) was used to code BCTs based on information presented in the included papers, as well as any published protocol papers. Two authors (CG, TC) independently coded BCTs for all included studies. Discrepancies were resolved through discussion.

The risk of bias of the studies included in the meta-analysis was assessed by CG and TC using the Cochrane Collaboration’s risk of bias tool [33]. Six different sources of bias were considered: selection bias, performance bias, detection bias, attrition bias, reporting bias, and other bias. Risk of bias was described as ‘low’, ‘high’ or ‘unclear’. Scoring conflicts were discussed and resolved by three authors (CG, TC, JS).

### Statistical methods

#### Between group differences in physical activity

Studies reporting total minutes of MVPA per week for control and experimental groups at baseline and post-intervention follow-up were included in a meta-analysis using Cochrane Review Manager 5.3 software [34]. Guidance for pooling data for meta-analysis recommends combining data that are as similar as possible, therefore trials reporting physical activity by other means (e.g. METs/wk., kcal/kg/day, walking time and kcal/week) were not included. The difference in MVPA mins/wk. between the control and intervention groups at the last post-intervention follow-up assessment were used to calculate effect size, and intention-to-treat data were used when available. When insufficient data were available for the purposes of meta-analysis (e.g. no standard deviation (SD) or unadjusted baseline data presented), authors were contacted to provide the data required. If standard errors or confidence intervals were presented instead of SD, we calculated the SDs. The vast majority of studies included a self-report measure of MVPA, therefore these data were used. Data from objective measures of physical activity were included if they were available. For studies reporting moderate and vigorous activity separately, combined MVPA and SDs were calculated using the following formula, as per [35]:

$$ {\overline{x}}_{MVPA}={\overline{x}}_{moderate\  PA}+{\overline{x}}_{vigorous\  PA} $$

To combine SDs, the following formula was used

$$ {\sigma}_{MVPA}=\sqrt{\left(\left({\sigma}_{moderate\  PA}^2\right)+\left({\sigma}_{vigorous\  PA}^2\right)\right)} $$

A random effects model was used to calculate standardized mean difference (SMD) with 95% confidence intervals. A random effects model was chosen as interventions and outcome measures of physical activity varied widely and this type of model is recommended where heterogeneity is suspected.

Post-hoc sensitivity analysis was performed to include only those studies with > 6 month follow-up data (a more conservative definition of maintenance of behaviour).

#### Pre-post change in physical activity within control group and intervention group

The change from baseline to post-intervention follow-up in minutes of MVPA per week within control and intervention groups was estimated separately in meta-analysis. As above, random effects models were used due to heterogeneity in interventions and outcomes measures.

Random effects models were also used to calculate mean difference with 95% confidence intervals for between and within group analysis to provide mean difference/change in MVPA mins/wk. to aid clinical interpretation.

Statistical approaches to explore associations between study population and intervention characteristics (including BCTs) and intervention effects e.g. meta-analysis, such as meta-regression, were considered. However, due to the relatively small number of included studies, these were not deemed feasible. Therefore, a narrative synthesis of these factors is presented using a similar categorisation methodology to Gardner et al. [36]. Trials were categorised as ‘very promising’, ‘quite promising’ or ‘not promising’. Category allocation was dependent on within or between group differences in physical activity behaviour at post-intervention follow-up. ‘Very promising’ trials included those with statistically significant between group differences in MVPA mins/wk. at post-intervention follow-up. ‘Quite promising’ were trials reporting within-group differences in the intervention group at post-intervention follow-up and ‘not promising’ were those that reported neither within nor between group differences at post-intervention follow-up. These categories are mutually exclusive. This categorisation enabled data interpretation and narrative synthesis of intervention and context, associated with the most and least promising interventions. In order to recognise intervention components that were associated with effectiveness, we identified BCTs that were common, defined as appearing in at least half, of the ‘very promising’ and ‘quite promising’ studies, and were uncommon, defined as appearing in less than half, of the ‘not promising’ studies.

## Results

Figure 1 shows the flow of studies for this review. Twenty seven studies met the inclusion criteria and were included in this review.

**Fig. 2:**
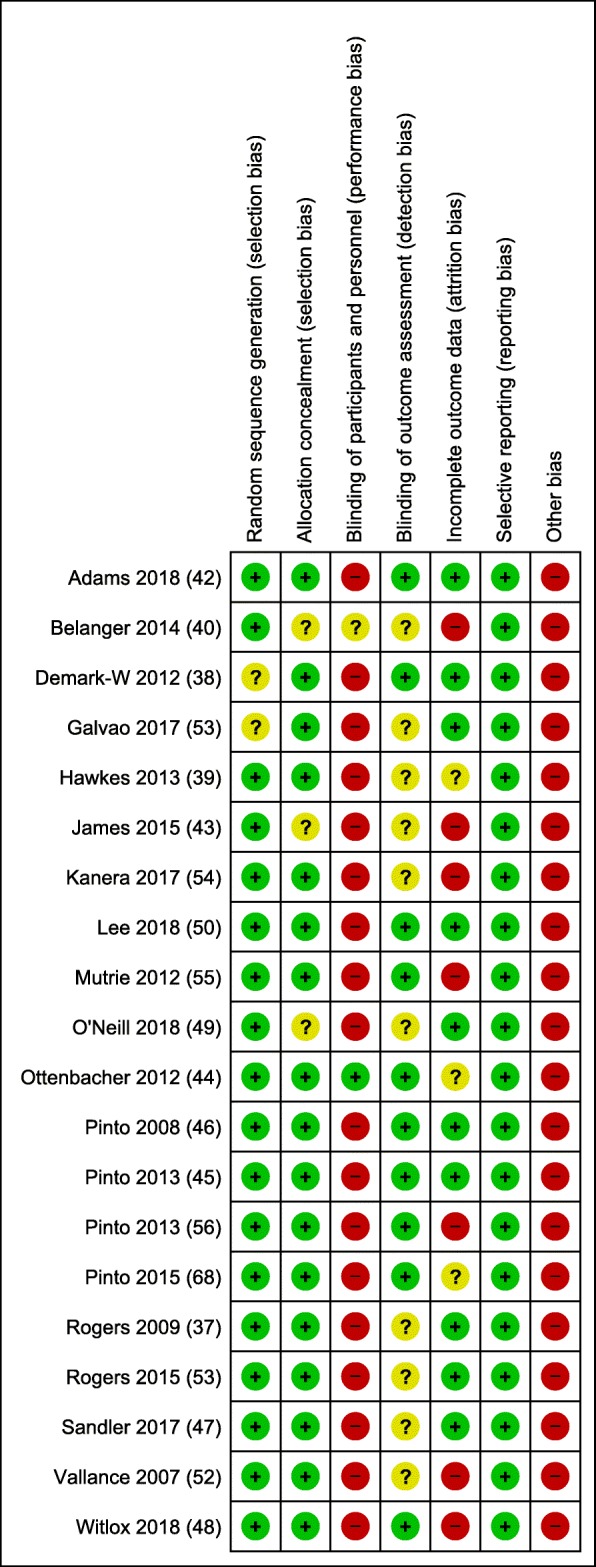
Risk of Bias Assessment

Figure 2 presents the risk of bias assessment of studies included in the meta-analyses. Random sequence generation and allocation concealment was reasonably well reported however the majority of studies did not demonstrate blinding of outcome assessments and a number were limited by attrition bias. Most studies were also judged as ‘high risk’ for other sources of bias with many using self-reported physical activity measures which is prone to bias.

Table 1 presents the characteristics of the populations from each study. Studies were published between 2006 and 2018 and more than half (15/27) were conducted in the USA and Canada. Sample sizes ranged from 41 [37] – 641 participants [38]. Most studies (18/27) included women diagnosed with breast cancer and participants were relatively young (average age 58.2 years) and well educated with 69.7% participating in some higher education. Number of comorbidities was infrequently reported and where data were available considerable variation was evident, ranging from more than 90% of participants reporting at least one comorbidity [39], to less than 30% reporting at least one comorbidity [40]. Table 2 provides data on intervention characteristics and context. Intervention modality varied from ‘light touch’ approaches such as providing printed materials only to more intensive designs including supervised exercise sessions and regular follow-ups, predominantly by telephone. The length of the interventions varied from a single contact [41] to 10 months of regular interactions [38]. Length of post-intervention follow-up ranged from 3 months to 5 years.

**Table 1:** Participant characteristics. Baseline physical activity and physical activity measure for studies included in meta-analysis

Authors and country	N	Cancer type	On treatment	Mean age	% female	% white	% higher education	Mean number of comorbidities	Exclusion based on current activity levels	Baseline physical activity (MVPA mins/wk) intervention group	Measure of physical activity
Very promising – Significant between group difference											
^a^ Mutrie et al. 2012 [55] UK	203	Breast cancer	Yes	51.6	100	NR	NR	NR	Excluded those who ‘regularly exercised’	367	Scottish physical activity questionnaire
Pinto et al. 2008 [46] USA	86	Breast cancer	No	^b^53.1	100	^b^95.5	^b^81.5	NR	Had to be relatively inactive < 20 mins vig or < twice a week of 30 mins moderate	82	7 Day Physical activity recall
Rogers et al. 2015 [57] USA	222	Breast	No	54.4	100	83.8	NR	2.2	Had to be relatively inactive < 30 mins vig or 60 mins mod PA	178	Actigraph
Rogers et al. 2009 [37] Canada	41	Breast	NR	53	100	93	NR	NR	Excluded if > 150 min mod or > 60 mins vig	96	Actirgraph
Belanger et al. 2014 [40] Canada	212	Breast, colorectal, other	Yes	18-39 yrs	60.8	85	67.5	28.3% > 1	Meta-synthesis Included only those from sub-analysis with baseline PA ≤ 300 mins/wk. at baseline	86	Godin Leisure Time exercise questionnaire
Pinto et al. 2015 [68] USA	76	Breast	Yes	55.6	100	98.7	89.5	NR	Had to be relatively inactive < 30 mins vig or < 90 mins mod PA	32	7 Day Physical activity recall
Pinto et al. 2013 [56] USA	192	Breast	No	^b^56	100	^b^94	^b^76	NR	Must be relatively inactive (< 30 mins vig or < 90 mins mod/wk)	49	7 Day Physical activity recall
Dhillon et al. 2017 [69] Australia	112	Lung	Yes	Median 64	45	NR	NR	NR			
Baumann et al. 2017 [70] Germany	194	Breast	No	^b^56	^b^100	NR	NR	NR			
Hawkes et al. 2013 [39] Australia	410	Colorectal	Yes	^b^66.3	^b^46.1	NR	NR	^b^91% ≥ 1	Had to include 1 poor health behaviour (< 150 mins MVPA/wk., < 2 servings of fruit or < 5 servings of veg, or overweight, BMI ≥ 25 kg/m^2^	59	Godin Leisure Time exercise questionnaire
Kanera et al., 2017 [54] Netherlands	462	Various (70% breast)	No	^b^55.9	^b^79.9	NR	31	0.35			
Witlox et al., 2018 [48] Netherlands	237	Breast and colon	Yes	50.8	91.1	NR	42	NR	No exclusions	879	SQUASH
Quite promising - Significant within-group difference baseline to post-intervention follow up											
Demark-Wahnefried et al. 2012 [38] USA	641	Breast, prostate and colorectal	No	^b^73	^b^55.3	^b^90	^b^61.9	NR	Baseline physical activity must be < 150 mins MVPA/wk	33.3	CHAMPS
Ottenbacher et al. 2012 [44] USA	400	Breast and Prostate	No	^b^57.6	59.5	85.5	89	2.15	Analysis of *N* = 400 in larger study were reported < 150 mins MVPA/wk	24	7 Day Physical activity recall
Vallance et al. 2007 [52] Canada	377	Breast	No	58	100	NR	NR	NR	No exclusions	119	Godin Leisure Time exercise questionnaire
Pinto et al. 2013 [45] USA	46	Colorectal	No	^b^57.6	^b^57	^b^98	^b^76.5	NR	Had to be relatively inactive < 60 mins of mod or < 20 mins vigorous	38	7-Day Physical activity recall
Leclerc et al. 2018 [71] Belgium	209	Breast	No	^b^53.4	100	NR	NR	NR			
Mayer et al. 2018 [72] USA	284	Colon	No	^b^58.6	^b^52	^b^89	^b^57	NR			
Lee et al. 2018 [50] Hong Kong	223	Colorectal	N	^b^65.2	^b^36.8	NR	^b^87.5	–	Unclear	498	Actigraph
Adams et al. 2018 [42] Canada	63	Testicular	N	43.7	0	90.5	NR	NR	excluded if performed ‘regular vigorous physical activity	125	Godin Leisure Time exercise questionnaire
Stolley et al. 2017 [73] USA	246	Breast	N	57.5	100	0	76	NR			
Not promising - No between or within group differences											
Galvo et al. 2017 [53] Australia	463	prostate	Yes	64.4	–	NP	62	NR	No exclusion based on PA	126	Godin Leisure Time exercise questionnaire
Nyrop et al. 2017 [41] USA	62	Breast	No	63.8	100	74	77	NR			
Carmack et al. 2006 [51] USA	134	Prostate	Yes	69.2	–	73	79	65.7% ≥ 2			
James et al. 2015 [43] Australia ^c^	108	Mixed	N	–	–	–	–	–	No exclusions	84	Active Australia survey
O’Neill et al. 2018 [49] Ireland	43	Esophagogastric	N	^b^65.7	^b^19	NR	NR	NR	No exclusions	132	Actigraph
Sandler et al. 2017 [47] Australia	46	Breast and colon	N	^b^51.2	^b^93.6	NR	NR	NR	No exclusions	30	IPAQ

*NR* not reported ^a^Used linked texts to extract some sample characteristics ^b^summed means across 2 groups ^c^demographic data not available as presented for both cancer patients and carers, *SQUASH* Short Questionnaire to Assess Health enhancing physical activity, *CHAMPS* Community Health Activities Model Program for Seniors, *IPAQ* International Physical Activity Questionnaire

**Table 2:** Intervention characteristics

Authors and country	Theoretical bases	PA intervention only	Supervised exercise	Intervention provider	Number of contacts	Setting and mode of delivery	Length of intervention	Length of follow-up	Control group instructions	Fidelity assessment	Recruitment rates	Drop out post-intervention follow up
Very promising – Significant between group difference												
^a^ Mutrie et al. 2012 [55] UK	SCT and TTM	Y	Y	Specifically trained exercise specialists	24	Group supervised exercise classes and home-based exercise with referral to GP exercise scheme	3 m	5 yrs	Printed recommendations. After 6 months offered personal ex plan and GP exercise scheme referral	N	1144/203 (18%)	Control = 59 (58%) Int = 57 (56%)
Pinto et al. 2008 [46] USA	TTM	Y	N	Researchers conducted phone calls	15	Telephone delivered home-based	6 m	9 m	Contact control - Cancer survivorship tip sheets (not PA related). Telephone calls as per intervention group to monitor symptoms	N	Cannot be calculated	Control = 4 (9.3%) Int =4 (9.3%)
Rogers et al. 2015 [57] USA	SCT	Y	Y	Exercise specialists and group facilitators trained by clinical psychologist	21	Supervised exercise sessions, counselling and group discussions. Home-based exercise encouraged	3 m	12 m	Usual care plus ACS written materials on PA recommendations	Y	Cannot be calculated	Control = 2 (3.6%) Int = 5 (4.5%)
Rogers et al. 2009 [37] Canada	SCT	Y	Y	Exercise specialist and clinical psychologist	21	Supervised exercise sessions and group discussion with home-based exercise encourage	3 m	6 m	Usual care plus ACS written materials on PA recommendations	Y	Cannot be calculated	Control = 3 (15%) Int = 2 (9.5%)
Belanger et al. 2014 [40] Canada	TPB	Y	N	-	0	Printed 11 chapter guidebook to promoted PA tailored to young adults	0 m	4 m	Non tailored print materials, 1 page handout	–	212/1908 (11%)	Control = 65 (61.3%) Int = 63 (59.4%)
Pinto et al. 2015 [68] USA	TTM and SCT	Y	N	Peer volunteers (with training)	16	Peer-led telephone consultation and mailed feedback reports	3 m	6 m	Contact control	N	76/291 (26%)	Control = 6 (16%) Int = 3 (7.7%)
Pinto et al. 2013 [56] USA	TTM and SCT	Y	N	No details provided	24	Health care professional advice plus telephone counselling plus printed materials	6 m	12 m	Health care professional advice plus contact control	Y	192/270 (71%)	Control = 5 (20.8%) Int = 6 (9.4%)
Dhillon et al. 2017 [69] Australia	TPB	Y	Y	Physical activity consultant	8	Weekly exercise sessions – home-based exercise was encouraged	2 m	6 m	Diet and PA education materials	N	112/254 (44%)	Control = 28 (50.9%) Int = 21 (37.5%)
Baumann et al. 2017 [70] Germany	None	Y	Y	Insufficient details	–	Residential rehab program with telephone follow-up	8 m	24 m	3 week rehab program but not FU care	N	No data provided	NP
Hawkes et al. 2013 [39] Australia	ACT	N	N	Health coaches with degrees in nursing, psychology or health promotion	15	Telephone counselling and printed materials	6 m	12 m	Generic printed materials promoting PA	Y	410/792 (52%)	Control = 42 (20.5%) Int = 44 (22.4%)
Kanera et al., 2017 [54] Netherlands	Intervention component derived from SCT, TPB, self-regulation theory and Integrated Model for Change	N	N	-	NR	Web-based lifestyle intervention	6 m	12 m	Waitlist control	N	518/1298 (40%)	Control = 19 (9.0) Int = 62 (36.7%)
Witlox et al., 2018 [48] Netherlands	SCT	Y	Y	Physiotherapist	36	Supervised and home-based physical activity	18wk	4 yr	Usual care but access to publically available programs after 18wks	N	237/503 (47%)	50.8% *n* = 60 control 41.2% *n* = 49 int
Quite promising - Significant within-group difference baseline to post-intervention follow up												
Demark-Wahnefried et al. 2012 [38] USA	SCT and TTM	N	N	Counsellors (no detail on training/expertise)	23	Personally tailored workbook, newsletters and telephone counselling	10 m	2 yrs	Waitlist control	N	641/20015 (3%)	Control = 77 (23.9%) Int = 76 (23.8%)
Ottenbacher et al. 2012 [44] USA	SCT	N	N	-	14	Tailored printed materials	10 m	2 yrs	Attention control – non tailored printed materials including promotion of PA	■ –	543/1570 (44%)	Control = 16 (6.5%) Int = 28 (14.1%)
Vallance et al. 2007 [52] Canada	No data provided	Y	N	No details provided	2	Recommendation to exercise, printed materials and pedometer	3 m	6 m	Recommendation to exercise	N	398/1590 (25%)	Control = 28 (29.2%) Int = 26 (27.9%)
Pinto et al. 2013 [45] USA	TTM and SCT	Y	N	Counsellors received training on theoretical basis	19	Telephone delivered home-based with printed materials	6 m	12 m	Contact control - Cancer survivorship tip sheets (not PA related). Telephone calls as per intervention group to monitor symptoms	Y	46/168 (27%)	Control = 3 (12%) Int = 1 (5%)
Leclerc et al. 2018 [71] Belgium	None described	Y	Y	Physiotherpaists and professor of physiotherapy and rehabilitation	36	Supervised group exercise and education	3 m	24 m	Control group – asked not to change exercise behaviour for the entire follow-up period	N	Cannot be calculated	Control = 55 (51.9%) Int = 53 (51.5%)
Mayer et al. 2018 [72] USA	SDT	Y	N	–	–	Smartphone App	6 m	9 m	Control Group – National Cancer Institute’s Facing Forward: Life after Cancer Treatment booklet and National Coalition for Cancer Survivorship’s Cancer Survival Toolbox + pedometer	–	284/465 (61%)	Data not reported at 9 m
^b^Lee et al. 2018 [50] Hong Kong	TPB and Health Action Process Approach	N	N	No details provided	32	Printed materials with motivational phone calls newsletters and group meetings	12 m	24 m	Usual care	N	Cannot be calculated	Control groups = 18 (16%) Int groups = 16 (14%)
Adams et al. 2018 [42] Canada	None described	Y	Y	No details provided	36	Supervised high intensity exercise sessions	3 m	6 m	Usual care – offered 6 week exercise training after final follow-up	N	7% of potentially eligible patient (63/948)	Control group = 5 (18%) Int groups 6 (17%)
Stolley et al. 2017 [73] USA	Grounded in a socioecological model	N	Y	Study trained community nutritionist and exercise trainer	48	Supervised Group exercise and education classes	6 m	12 m	Self-guided weight management intervention – printed materials only	N	246/448 (55%)	Control = 21 (17%) Int = 18 (14%)
Not promising - No between or within group differences												
Galvo et al. 2017 [53] Australia	None described	Y	N	Trained peer support workers	6	Self-management materials and telephone-based group peer support	6 m	12 m	Published patient education materials	N	463/1314 (32%)	Control = 37 (19.1%) Int = 47 (25.5%)
Nyrop et al. 2017 [41] USA	None described	Y	N	-	1	Workbook and home-based walking	6 wks	6 m	Waitlist control	–	78/344 (23%)	*N* = 21 (33.9%) whole sample
Carmack et al. 2006 [51] USA	SCT and TTM	Y	N	Group facilitators, supervised by clinical psychologist	20	Group counselling, home-based exercise encouraged	6 m	12 m	Standard care	Y	Cannot be calculated	Control = 3 (8.1) Int = 11 (23.9%) Education control = 7 (13.7%)
James et al. 2015 [43] Australia ^c^	SCT	N	Y	Exercise physiologist	6	Group sessions and workbook	8wk	20wks	Waitlist control	N	-	-
O’Neill et al. 2018 [49] Ireland	Non described	N	Y	A multidisciplinary team	14	Supervised and home-based exercise with education	3 m	6 m	Usual care	N	43/264 = 16%	Control = 1 (5%) Int = 2 (9%)
Sandler et al. 2017 [47] Australia	CBT	N	N	Exercise physiologist and clinical psychologist	5	Manualised exercise programme with face-to-face consultations	3 m	6 m	Education package and 1 face-to-face meeting with exercise professionals	N	55/46 = 84%	Control = 2 (8%) Int = 4 (18%)

^a^Used linked texts to extract some intervention characteristics ^b^ Study included 4 groups, comparison group classed as usual care or diet intervention only, intervention group = PA only or PA plus diet ^c^ attrition data not available as presented for both cancer patients and carers, *TTM* Transtheoretical Model, *SCT* Social Cognitive Theory, *CBP* Cognitive Behavioural Therapy, *SDT* Self-Determination Theory, *ACT* Acceptance Commitment Therapy, *TBP* Theory of Planned Behaviour

After obtaining data from authors of 7 of the included studies [42–48] 19 studies were eligible to be included in the between group meta-analysis; of these, 12 studies excluded participants who were already meeting physically active guidelines, however the median baseline levels of physical activity in the intervention groups was 86 mins/wk. of MVPA, range 23.5 (44)-879 [48].

Two of the included studies presented physical activity data on a subsample of participants. Ottenbacher et al. [44] presented analysis for participants in the FRESH START trial who failed to meet physical activity guidelines (< 150 mins MVPA/wk) on study entry: 400 of the 543 breast and prostate cancer survivors enrolled in this home-based lifestyle intervention. Belanger et al. [40] provided data for the 96 of the 212 young adult cancer survivors who were engaging in ≤300 min of physical activity at baseline. These data were included in the meta-analysis.

### Behaviour change outcomes

**Fig. 3:**
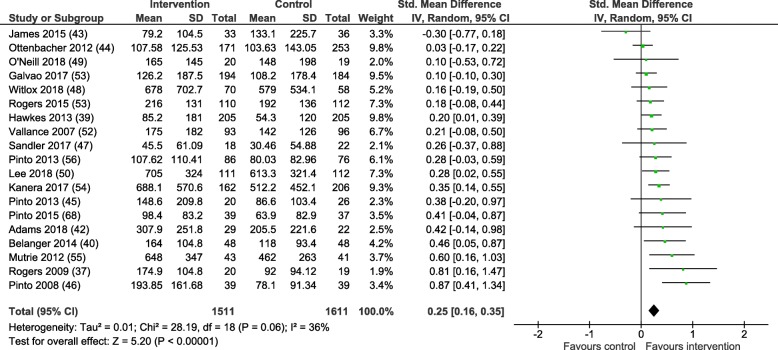
Between group difference in MVPA mins/wk. at post-intervention

Nineteen of the 27 included studies provided PA data as MVPA mins/wk. data for the experimental and control groups at the post-intervention follow-up and were included in the meta-analysis. All but three studies presented data on self-reported levels of physical activity, Rogers et al. [37], O’Neil et al. [49] and Lee et al. [50] reported only objectively measured physical activity using accelerometry. Two studies included more than one intervention group [51, 52]. In both instances the most intensive intervention was compared to the control group in meta-analyses. Figure 3 presents a forest plot detailing the SMD with 95% confidence intervals (CIs) for the post-intervention MVPA mins/wk. data. The SMD between groups favoured the intervention group with a small estimated effect (0.25; (95% CI = 0.16–0.35)) and moderate statistical heterogeneity (I^2^ = 36%). The mean difference between the intervention and controls groups was 39.88 (95% CI = 22.78-56.97) MVPA mins/wk., *p* < 0.01.

#### Post-hoc sensitivity analysis

Post-hoc sensitivity analysis was conducted with the 10 studies that presented data on MVPA mins/wk. a minimum of 6 months after intervention completion [39, 44, 45, 48, 50, 53–57]. A very similar SMD was found; 0.21; 95% CI = 0.12–0.29, p < 0.01, I^2^ = 13%.

#### Within group meta-analysis

**Fig. 4:**
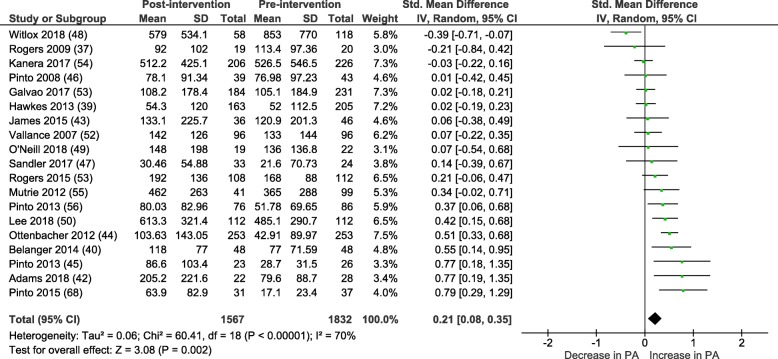
Control group change in MVPA mins/wk. from baseline to post-intervention

Within group pre-post intervention analyses of physical activity behaviour in control groups reveals a small SMD:  0.21 (95% CI = 0.08-0.35), *P* < 0.01, I^2^ = 70%. See Fig. 4. Mean difference =  27.48 (95% CI = 11.48-43.49) mins/wk. of MVPA, p < 0.01.

**Fig. 5:**
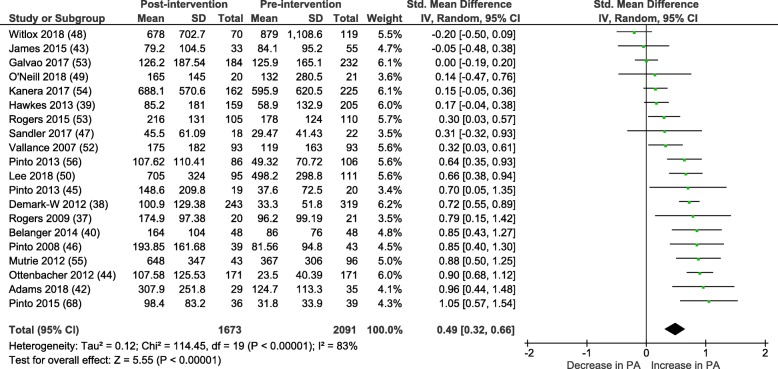
Intervention group change in MVPA mins/wk. from baseline to post-intervention

Within group analyses of physical activity behaviour in the intervention groups revealed a moderate SMD 0.49 (95% = CI 0.32–0.66), p < 0.01, with high heterogeneity (I^2^ = 83%) as shown in Fig. 5. The mean difference between baseline and post-intervention follow-up was 65.30 (95% CI = 45.59–85.01) mins/wk. of MVPA. Of note, one additional trial was included in this analysis [38]. Demark-Wahnefried et al. (2012) provided baseline and post-intervention (2 year) follow-up for the intervention group. The comparison group were offered the intervention at 1 year and were therefore excluded from all other meta-analyses.

As the correlation between objective and self-reported physical activity levels is often poor, all meta-analyses were re-run in a post-hoc sensitivity analysis removing the 3 studies that used objective measures of physical activity; this had negligible impact on the results: between group SMD: 0.27 (95% CI 0.15–0.38) I^2^ = 46%, within group pre-post analysis for control groups SMD: 0.23 (95% CI 0.09–0.38) I^2^ = 70%, within group pre-post analysis for intervention groups SMD: 0.48 (95% CI 0.29–0.67) I^2^ = 85%.

### BCTs and physical activity outcomes

Studies included an average of 10.3 BCTs (range 2–20), goal setting (*n* = 25), self-monitoring (*n* = 22), instruction on how to perform a behaviour (*n* = 19) and problem solving (*n* = 18) were most frequently reported (see Table 3). There were few notable differences in the BCTs identified within studies when comparing across the classifications ‘very promising’, ‘quite promising’ or ‘not promising’. The BCTs of ‘goal setting’, ‘problem solving’, ‘self-monitoring’ and ‘instruction on how to perform a behaviour’ were used often, irrespective of study effectiveness. Additionally the BCTs ‘action planning’, ‘graded tasks’ and ‘social support (unspecified)’ were present in the ‘very promising’ and ‘quite promising’ studies but most often absent from the ‘not promising’ studies. When considering the study populations and characteristics as described in Table 1, participants in the studies classified as ‘not promising’ tended to be older (mean age 63 yrs. versus 57 yrs. in the ‘very promising’ and ‘quite promising’ studies). Also, supervised physical activity tended not to be provided as part of the intervention in the former studies. There were also fewer contacts with participants during the intervention. Moreover, two of the studies [41, 47] purposefully recruited participants with limitations, pain and fatigue respectively, and two included exclusively prostate cancer patients, many of whom were still undergoing treatment.

**Table 3:** Behaviour change techniques identified in each intervention

		Very promising (significant between group difference at post intervention follow-up)													Quite promising (significant change from baseline to post intervention follow-up)								Not promising (no significant between or within group differences)						
BCT no.	BCT label	Baumann et al (71)	Kanera et al (54)	Rogers et al (57)	Pinto et al (69)	Hawkes et al (39)	Mutrie et al (55)	Rogers (37)	Pinto et al (56)	Dhillon et al (70)	Pinto et al (46)	Witlox et al (48)	Stolley et al (74)	Belanger et al (40)	Demark-Wahnefried (38)	Ottenbacher et al (44)	Vallance et al (52)	Leclerc et al (72)	Lee et al (50)	Adams et al (42)	Mayer et al (73)	Pinto et al (45)	Galvo et al (53)	James et al (43)	Carmack et al (51)	O’Neil et al (49)	Sandler et al (47)	Nyrop et al (41)	Total number of studies using BCT
1.1	Goal setting (behaviour)		1	1	1	1	1	1	1	1	1	1	1	1	1	1	1	1	1	1	1	1	1		1	1	1	1	25
1.2	Problem solving		1	1	1	1		1	1	1	1		1	1	1	1	1					1	1	1	1			1	18
1.3	Goal setting (outcome)	1				1				1																			3
1.4	Action planning		1	1	1	1	1	1	1	1	1	1		1			1					1			1			1	15
1.5	Review behaviour goal(s)		1	1	1	1						1	1												1		1		8
1.6	Discrepancy between current behaviour and goal		1												1	1													3
1.9	Commitment					1																							1
2.2	Feedback on behaviour		1	1	1				1			1			1	1					1	1	1		1				11
2.3	Self-monitoring of behaviour		1	1	1	1		1	1	1	1	1	1	1	1	1	1		1		1	1		1	1	1	1	1	22
2.6	Biofeedback				1																								1
2.7	Feedback on outcome(s) of behaviour																						1						1
3.1	Social support (unspecified)	1	1	1	1	1	1	1	1	1	1	1	1		1		1	1	1	1	1	1	1		1				18
3.2	Social support (practical) staff						1														1								2
4.1	Instruction on how to perform a behaviour		1	1	1	1	1	1	1	1	1	1	1	1	1	1	1					1		1	1			1	19
4.2	Information about antecedents					1																							1
5.1	Information about health consequences			1		1	1	1	1	1			1	1		1	1	1			1	1		1	1			1	16
5.2	Salience of consequences			1				1	1																				3
5.3	Information about social and environmental consequences		1							1																			2
5.6	Information about emotional consequences			1																								1	2
6.1	Demonstration of behaviour			1				1		1				1			1				1				1				7
7.1	Prompts/cues					1			1		1		1		1	1					1	1							8
8.1	Behavioural practice/rehearsal			1			1			1															1				4
8.6	Generalisation of a target behaviour			1			1	1		1															1				5
8.7	Graded tasks		1	1	1			1	1		1				1	1	1		1	1		1				1	1		14
9.1	Credible source		1	1	1	1	1	1	1	1		1			1	1	1	1			1		1	1	1	1		1	19
9.2	Pros and cons		1							1															1				3
10.4	Social reward								1		1				1	1						1			1				6
11.2	Reduce negative emotions			1		1		1									1						1		1				6
12.5	Adding objects to the environment			1	1	1			1	1	1		1		1	1	1		1		1	1	1	1	1		1		17
13.2	Framing/reframing			1																					1				2
15.1	Verbal persuasion about capabilities			1		1		1	1		1	1				1			1			1						1	10
	Total No. BCTs	2	13	20	12	16	9	15	15	16	11	9	9	7	12	13	12	4	7	3	10	13	9	6	18	4	5	9	

Mayer et al. report changes to the intervention during the study so not all BCTs would have been available to all participants

When considering physical activity behaviour change in the control groups, four of the eight studies describing significant increases in physical activity from baseline to post intervention follow-up included ‘attention control’ compared, to only one study that used attention control but did not find any significant change.

## Discussion

This is the first systematic review and meta-analysis to synthesise the evidence from RCTs examining long-term physical activity behaviour change following intervention in cancer populations. With a significant overall SMD (0.25; 95% CI = 0.16–0.35) in MVPA mins/wk. we can conclude interventions achieved a small effect, compared with controls, on long-term physical activity behaviour defined as a minimum 3 month follow-up. Results were similar for studies presenting a minimum 6 month follow-up period. Also unique to this review is the synthesis of evidence of physical activity behaviour change in the control groups with an average increase of approximately 30 mins/wk. MVPA from baseline to post intervention follow-up. This suggests that the standard mean differences between the intervention and control groups may underestimate the impact of the interventions on physical activity behaviour. Indeed, when considering data only from the intervention groups, we saw an average increase of 65.30 (95% CI = 45.59–85.01) mins/wk. of MVPA at the last follow-up point. Such a change, especially in the intervention groups, may be clinically meaningful. There is evidence that older men who move from being sedentary to engaging in at least light activity have significantly lower risk of all-cause mortality than those who remain sedentary [58]. Furthermore, there is data showing that women with breast cancer who engage in 3–8.9 MET-hrs/wk. of physical activity, had a relative risk of death from breast cancer of 0.50 (95% CI, 0.31–0.82) compared to 0.80 (CI 0.60–1.06) for those doing less that 3 MET-hours per week, with 3 MET hours equivalent to walking at an average pace of 2 to 2.9 mph for 1 h [59].

The notion of contamination in control groups in lifestyle interventions is a widely acknowledged limitation of such trials. Steins Bisschop et al. [28] conducted a systematic review of control group design, contamination and drop out in oncology trials. They found that 75% of studies in their review reported control group contamination. Furthermore, Waters and colleagues [60] found that 28% of physical activity interventions reviewed (*n* = 28, which are not limited to cancer populations) reported meaningful improvements in physical activity levels of the control groups. However the current review is the first to quantify the degree of this change across cancer trials. Notably, we showed that 7 of the 19 studies (35%) showed statistically significant improvements in physical activity in control groups at follow-up.

By identifying the BCTs included within the studies we hoped to unpick the key ingredients that may lead to successful, long-term behaviour change in cancer populations. ‘Goal setting (behaviour)’, ‘self-monitoring of behaviour’, ‘problem solving’ and ‘instruction on how to perform a behaviour’ were frequently reported across studies, irrespective of effectiveness. There were few clear differences in BCTs identified in the ‘very promising’ and ‘quite promising’ verses the ‘not promising’ studies. The exceptions were; 1) ‘graded tasks’, setting easy tasks and make them increasingly difficult but achievable. 2) ‘social support (unspecified)’, that is, providing or arranging social support or non-contingent praise or reward for performance of the behaviour; this includes studies that used motivational interviewing techniques. 3) ‘action planning’, encourage detailed planning of the behaviour e.g. where and when you plan to exercise. A recent review of maintenance of weight loss after cancer report similar findings [61]. Specifically, Hoedjes et al. found that the BCTs of ‘goal setting’, ‘action planning’, ‘social support’ and ‘instruction of how to perform the behaviour’ were present in interventions that effectively promoted sustained weight loss. However the authors did not describe the BCTs used in the unsuccessful interventions and therefore no comparisons are made. Similarities can also be seen in a recent review of maintenance of physical activity behaviour change in inactive healthy adults, with a small effect on physical activity behaviour at six months follow-up (d = 0.21, 95% CI = 0.12–0.30). Effectiveness was associated with ‘action planning’, ‘instruction on how to perform the behaviour’, ‘prompts/cues’, ‘behaviour practice/rehearsal’, ‘graded tasks’ and ‘self-reward’ [62].

It appears that inclusion of certain BCTs may increase the likelihood of intervention success, however the striking similarities of BCTs across ‘very promising’, ‘quite promising’ and ‘not promising’ studies in the current review suggests there are other population or context characteristics that impact on effectiveness.

In general, across all included studies, participants were relatively young, female, well-educated and predominantly white. Recruitment rates were variable but typically low, suggesting an amotivated population and/or strict inclusion/exclusion criteria. It appears therefore that current interventions reflect and reinforce structural societal inequalities. Participants in the studies that did not produce any notable change in behaviour tended to be older, two comprised exclusively prostate cancer populations and two included those with pain and clinically significant fatigue. As such, it may be that a stepped care approach to behaviour change is needed. This finding is supported by Morey and colleagues [63] who performed group trajectory analysis for patients participating in the RENEW study, a distanced-based multimodal lifestyle interventions in older long-term cancer survivors. They found that patients who remained inactive throughout the study had low levels of physical function at baseline which continued to decline over time. This is in contrast to those who achieved marked improvement in physical activity throughout the 12-month intervention and 12-month follow-up periods who reported considerably higher levels of physical function on study entry. By providing stratified support, offering more intensive interventions and one-to-one support to those who need it most, a more representative population of cancer patients may be encouraged to engage in positive behaviour change which will be more likely to affect change. Such an approach is supported by a recent Individual Patient Data meta-analysis examining the moderator effect of baseline values on the exercise outcomes of fatigue, aerobic fitness, muscle strength, quality of life and physical function [64]. Moreover, Buffart and colleagues found that for patients who had completed cancer treatment, those with worst baseline QoL, fatigue and physical function experienced the largest improvements following exercise intervention, suggesting the greatest impact of interventions may be seen by targeting those most in need.

Evidence presented here suggests that motivated, well-educated, younger and white patients may achieve a clinically important increase in their long-term physical activity behaviour with a relatively low-intensity intervention. This is supported by the observation of increases in physical activity in the control groups who were typically provided with brief written information. Nonetheless, inclusion of a supervised component and frequent contact with participants may further increase intervention effectiveness. This is corroborated by the finding that four of the eight studies that found significant improvements in physical activity within the control group’s used a contact control study design, so frequent contact may have prompted participants to become active. Similar conclusions were drawn in a recent review by Bluethmann et al. [18]. In their synthesis of 14 RCTs aimed at increasing physical activity in breast cancer survivors they explored the effect of intervention intensity (i.e. number of intervention sessions) on behaviour change. They found that higher intensity interventions tended to produce larger effects, but some of the largest effects came from interventions they had categorised as ‘medium’ intensity, including home-based programmes with telephone support. This is supported by an earlier review of broad reach interventions, concluding telephone, print and web-based interventions were effective in initiating behaviour change [31]. However, both reviews found limited evidence for maintenance of change. Finally, in a review of maintenance of behaviour change, although not limited to cancer populations, Fjeldsoe et al. reported that physical activity and dietary interventions with more intervention contacts were more likely to achieve maintenance of behaviour change.

Another important consideration is that participants choosing to enrol in the physical activity interventions tended to already be engaging in some physical activity. Of the 19 studies included in the between groups meta-analysis, 12 excluded participants who were already meeting physically active guidelines; however the median baseline levels of physical activity in the intervention groups was 86 mins/wk. of MVPA. It is likely that mechanisms of behaviour change are different for those who are engaging in no physical activity at all versus those who are somewhat active. As such alternative intervention methods may be required if targeting the more inactive and sedentary populations. This is of utmost importance given that population health benefits may be achieved by supporting those who are sedentary to becoming moderately active [65]. Also, the dose response relationship between physical activity and health benefits, particularly in cardiovascular disease, supports the message that ‘some physical activity is better than none’ [66] and older adults who participate in any amount of physical activity will see some health benefits [67].

It is important that the results of this paper are interpreted with some caution. The meta-analysis relied almost exclusively on data collected from self-reported physical activity measures, which are known to have poor correlation with objective measures of physical activity. There was also considerable variation in outcome measures used across studies. This may explain in part the wide range in baseline levels of physical activity and high heterogeneity reported in the within-group meta-analysis. When considering conclusions regarding BCTs associated with effectiveness, these findings are limited by the incomplete reporting of interventions and their components. Furthermore, the guidelines for coding of BCTs are very stringent so it is possible that BCTs embedded in interventions are not always captured by the coding if descriptions are not sufficiently precise. When describing interventions in future studies, we suggest authors refer to Michie et al’s [22] coding scheme to ensure BCTs are appropriately documented and thus accurately coded in future efforts of data synthesis. Finally, it is possible that our method of trial classification as ‘very promising’, ‘quite promising’ or ‘not promising’ may result in false negatives if studies are not powered to detect statistical differences in physical activity.

## Conclusions

This is the first systematic review and meta-analysis of physical activity maintenance across cancer types. When considering differences between intervention and comparison groups, small differences are evident in favour of intervention groups. Improvements in physical activity behaviour in control groups suggest these between group analysis may underestimate intervention effect. Meta-analysis of change in activity levels in intervention groups indicates a clinically significant mean increase of over 1 h per week at post intervention follow-up. Analysis of intervention components and context suggests reasonably low-intensity interventions may be sufficient in prompting lasting behaviour change in motivated, young, well educated and white populations but that more intensive support is likely to be required for other populations, especially for older people and those with physical limitations. Future interventions should seek to encourage engagement from more representative samples including older adults, those from ethnic minorities and less educated backgrounds. A stepped care approach to intervention design and delivery may enable effective use of limited resources with additional support provided to those most in need.

## Additional file

Additional file 1: Medline search strategy. (DOCX 18 kb)
