# Supplementary material for: Systematic review and meta-analysis of maintenance of physical activity behaviour change in cancer survivors
Source: Int J Behav Nutr Phys Act. 2019 Apr 27;16:37. doi: 10.1186/s12966-019-0787-4 (PMC6486962; doi:10.1186/s12966-019-0787-4)
Supplement: Supplementary file 1 — Medline search strategy. (DOCX 18 kb) [file 12966_2019_787_MOESM1_ESM.docx]

| Database, Host, Dates Searched | Search Strategy | Results |
| --- | --- | --- |
| Database: Ovid **MEDLINE**(R) Epub Ahead of Print, In-Process & Other Non-Indexed Citations, Ovid MEDLINE(R) Daily and Ovid MEDLINE(R) <1946 to Present>  Searched 15/08/2018 | 1 cancer surviv*.mp. (17875)  2 cancer patient*.mp. (157239)  3 1 or 2 (171789)  4 exp Exercise/ or exp Exercise Therapy/ (190972)  5 (accelerometer* or pedometer*).tw,kw. (12020)  6 (prehabilitat* or "pre-habilitat*" or "pre habilitat*").tw,kw. (249)  7 (aerobics or isometric* or pylometric*).tw,kw. (32755)  8 exercis*.tw,kw. (262398)  9 exp Exercise Movement Techniques/ (6883)  10 ("weight bearing" or "weight lifting").tw,kw. (13472)  11 "resistance training".tw,kw. (5930)  12 (physical adj2 (exertion or training or fitness or movement or activity or activities or endurance or condition*)).tw,kw. (110039)  13 ("physically fit" or "physically active").tw,kw. (7717)  14 (graded adj2 activit*).tw,kw. (431)  15 (strength adj2 training).tw,kw. (4596)  16 rehabilitat*.tw,kw. (146410)  17 endurance.tw,kw. (26138)  18 exp Walking/ (45826)  19 walk*.tw,kw. (99098)  20 exp Physical Fitness/ (26462)  21 Physical Endurance/ (18731)  22 Physical Exertion/ (58897)  23 exp Sports/ (164740)  24 sport*.tw,kw. (62673)  25 exp Running/ (18303)  26 (running or jogging).tw,kw. (53235)  27 or/4-26 (791864)  28 3 and 27 (5855)  29 pilot*.tw,kw. (133799)  30 (intervention* or "post-intervention" or program* or workbook* or "work book*").tw,kw. (1440494)  31 "Outcome Assessment (Health Care)"/ (63603)  32 "Outcome and Process Assessment (Health Care)"/ or Patient Reported Outcome Measures/ (26008)  33 trajectory.tw,kw. (30243)  34 project*.tw,kw. (289426)  35 (strategy or strategies).tw,kw. (834307)  36 "process evaluation".tw,kw. (2558)  37 (feasibilit* or feasabilit*).tw,kw. (146415)  38 (pretest* or "pre-test*" or "pre test*").tw. (21552)  39 "pretest-posttest".tw. (2575)  40 (posttest* or "post-test*" or "post test*").tw. (19035)  41 (pre- adj5 post).ab. (71305)  42 trial*.ti. (250107)  43 exp Clinical Trial/ (822725)  44 randomized controlled trial.pt. (475615)  45 controlled clinical trial.pt. (96073)  46 clinical trials as topic.sh. (189103)  47 Evaluation Studies/ (235855)  48 Validation Studies/ (88275)  49 evaluation studies as topic/ or feasibility studies/ or pilot projects/ or program evaluation/ or validation studies as topic/ (335743)  50 Research Design/ (97707)  51 exp Telemedicine/ (22455)  52 (telemedicine or telehealth or mhealth or ehealth).tw,kw. (15238)  53 or/29-52 (3922108)  54 28 and 53 (3601)  55 (behavio?r and chang* and exercis* and cancer and survivor*).mp. [mp=title, abstract, original title, name of substance word, subject heading word, keyword heading word, protocol supplementary concept word, rare disease supplementary concept word, unique identifier, synonyms] (193)  56 (lifestyle* and chang* and exercis* and cancer and survivor*).mp. (155)  57 (physical* and activit* and chang* and exercis* and cancer and survivor*).mp. (283)  58 or/55-57 (388)  59 54 or 58 (3682)  60 limit 59 to english language (3516) | 3518 |
|  |  |  |
| Database: Ovid **Embase** <1974 to 2017 August 14>  Searched 15/08/2017 | 1 exp cancer survivor/ or cancer survivor*.mp. (23847)  2 exp cancer patient/ or cancer patient.mp. (214210)  3 or/1-2 (233447)  4 exp exercise/ (282542)  5 exp physical activity/ (329158)  6 exercis*.tw,kw. (333794)  7 (accelerometer* or pedometer*).tw,kw. (14666)  8 (prehabilitat* or "pre-habilitat*" or "pre habilitat*").tw,kw. (341)  9 (aerobics or isometric* or pylometric*).tw,kw. (36799)  10 exp kinesiotherapy/ (64949)  11 "weight bearing".tw,kw. (15932)  12 "weight lifting".tw,kw. (1051)  13 "resistance training".tw,kw. (7080)  14 (physical adj2 (exertion or training or fitness or activity or activities or endurance)).tw,kw. (135462)  15 ("physically fit" or "physically active").tw,kw. (9298)  16 (graded adj2 activit*).tw,kw. (518)  17 (strength adj2 training).tw,kw. (5985)  18 (yoga or pilates or "tai chi").tw,kw. (6818)  19 endurance.tw,kw. (31908)  20 walking/ (55910)  21 walk*.tw,kw. (129406)  22 exp sport/ (133895)  23 running/ (21406)  24 jogging.tw,kw. (1815)  25 jogging/ (1687)  26 or/4-25 (879592)  27 3 and 26 (7745)  28 (pilot* or project* or scheme* or campaign* or initiative* or strateg*).tw,kw. (1754515)  29 (intervention* or "post-intervention" or program* or workbook* or "work book*").tw,kw. (1840989)  30 outcome assessment/ (387352)  31 "patient reported outcome measure*".tw,kw. (3552)  32 trajectory.tw,kw. (34787)  33 "theory of planned behavio?r".tw,kw. (2808)  34 "message framing".tw,kw. (231)  35 "process evaluation".tw,kw. (2828)  36 (feasibilit* or feasabilit*).tw,kw. (192088)  37 (pretest* or "pre-test*" or "pre test*").tw. (27589)  38 "pretest-posttest".tw. (2719)  39 (posttest* or "post-test*" or "post test*").tw. (24234)  40 (pre- adj5 post).ab. (119191)  41 trial.ti. (233031)  42 exp clinical trial/ (1240734)  43 randomized controlled trial/ (466650)  44 evaluation study/ or program evaluation/ (36925)  45 validation study/ (65793)  46 feasibility study/ (82060)  47 pilot study/ (113927)  48 methodology/ (1658530)  49 study.ti. (1387249)  50 *"randomized controlled trial (topic)"/ (4287)  51 "systematic review"/ (146472)  52 intervention study/ (33799)  53 exp telehealth/ (31202)  54 (telemedicine or telehealth or teleconsultation or mhealth or ehealth).tw,kw. (18148)  55 or/28-54 (7240281)  56 27 and 55 (5362)  57 limit 56 to english language (5252)  58 cancer risk/ or cancer screening/ or cancer prevention/ (222316)  59 57 not 58 (4918)  60 limit 59 to (conference abstracts or medline) (2397)  61 59 not 60 (2521) | 2521 |
|  |  |  |
| **Web of Science**  Science Citation Index Expanded (SCI-EXPANDED)  Conference Proceedings Citation Index- Science (CPCI-S)  Conference Proceedings Citation Index- Social Science & Humanities (CPCI-SSH)  Conference Abstract search 2015-current  Searched on 15/08/2017 | # 1 38,630 (TS=("cancer survivor*" or "cancer patient*")) AND LANGUAGE: (English)  # 2 47,987 (TS=(exercis*))  # 3 40,069 (TS=(physical NEAR (activity or activities or exertion or training or fitness or movement or endurance or condition*)))  # 4 1,396 (TS=("physically fit" or "physically active"))  # 5 8,143 (TS=(acceleromet* or pedometer*))  # 6 154 (TS=(prehabilitat* or "pre-habilitat*" or "pre habilitat*"))  # 7 4,615 (TS=(aerobics or isometric* or plyometric*))  # 8 1,650 (TS=("weight bearing" or "weight lifting"))  # 9 1,525 (TS=("resistance training"))  # 10 864 (TS=("strength training"))  # 11 1,245 (TS=(yoga or pilates or "tai chi"))  # 12 6,990 (TS=(endurance))  # 13 88,165 (TS=(walking or "walk test*" or jogging or running ))  # 14 169,581 #13 OR #12 OR #11 OR #10 OR #9 OR #8 OR #7 OR #6 OR #5 OR #4 OR #3 OR #2  # 15 1,678 #14 AND #1  # 16 160 #14 AND #1  Refined by: DOCUMENT TYPES: ( MEETING ABSTRACT OR PROCEEDINGS PAPER ) AND LANGUAGE: (English) | 160 |
|  |  |  |
| Psychinfo  Searched: 16/08/2017 | S43 S31 OR S41 Narrow by Language: - english  Search modes - Find all my search terms Interface - EBSCOhost Research Databases  Search Screen - Advanced Search  Database - PsycINFO 2,118  S42 S31 OR S41 Search modes - Find all my search terms Interface - EBSCOhost Research Databases  Search Screen - Advanced Search  Database - PsycINFO 2,118  S41 S3 AND S40 Search modes - Find all my search terms Interface - EBSCOhost Research Databases  Search Screen - Advanced Search  Database - PsycINFO 386  S40 S32 OR S33 OR S34 OR S35 OR S36 OR S37 OR S38 Search modes - Find all my search terms Interface - EBSCOhost Research Databases  Search Screen - Advanced Search  Database - PsycINFO 877  S39 postsurgical and physical and activit* Search modes - Find all my search terms Interface - EBSCOhost Research Databases  Search Screen - Advanced Search  Database - PsycINFO 96  S38 postsurgical and exercis* Search modes - Find all my search terms Interface - EBSCOhost Research Databases  Search Screen - Advanced Search  Database - PsycINFO 38  S37 postsurgical and rehabilitat* Search modes - Find all my search terms Interface - EBSCOhost Research Databases  Search Screen - Advanced Search  Database - PsycINFO 116  S36 physical and activit* and intervention* and survivor* Search modes - Find all my search terms Interface - EBSCOhost Research Databases  Search Screen - Advanced Search  Database - PsycINFO 476  S35 physical and activit* and behavio?r* and survivor* Search modes - Find all my search terms Interface - EBSCOhost Research Databases  Search Screen - Advanced Search  Database - PsycINFO 81  S34 lifestyle* and intervention* and behavio?r* and survivor* Search modes - Find all my search terms Interface - EBSCOhost Research Databases  Search Screen - Advanced Search  Database - PsycINFO 16  S33 lifestyle* and chang* and survivor* Search modes - Find all my search terms Interface - EBSCOhost Research Databases  Search Screen - Advanced Search  Database - PsycINFO 188  S32 behavio?r and chang* and survivor* Search modes - Find all my search terms Interface - EBSCOhost Research Databases  Search Screen - Advanced Search  Database - PsycINFO 99  S31 S3 AND S30 Search modes - Find all my search terms Interface - EBSCOhost Research Databases  Search Screen - Advanced Search  Database - PsycINFO 2,045  S30 S4 OR S5 OR S6 OR S7 OR S8 OR S9 OR S10 OR S11 OR S12 OR S13 OR S14 OR S15 OR S16 OR S17 OR S18 OR S19 OR S20 OR S21 OR S22 OR S23 OR S29 Search modes - Find all my search terms Interface - EBSCOhost Research Databases  Search Screen - Advanced Search  Database - PsycINFO 337,954  S29 S24 AND S28 Search modes - Find all my search terms Interface - EBSCOhost Research Databases  Search Screen - Advanced Search  Database - PsycINFO 804  S28 S25 OR S26 OR S27 Search modes - Find all my search terms Interface - EBSCOhost Research Databases  Search Screen - Advanced Search  Database - PsycINFO 67,835  S27 ("after cancer" or "with cancer") Search modes - Find all my search terms Interface - EBSCOhost Research Databases  Search Screen - Advanced Search  Database - PsycINFO 67,832  S26 "following cancer" Search modes - Find all my search terms Interface - EBSCOhost Research Databases  Search Screen - Advanced Search  Database - PsycINFO 165  S25 DE "Survivors" and cancer Search modes - Find all my search terms Interface - EBSCOhost Research Databases  Search Screen - Advanced Search  Database - PsycINFO 4,237  S24 DE "Behavior Change" OR DE "Behavior Modification" OR DE "Behavior Therapy" OR DE "Behavioral Assessment" Search modes - Find all my search terms Interface - EBSCOhost Research Databases  Search Screen - Advanced Search  Database - PsycINFO 49,032  S23 TX running or jogging Search modes - Find all my search terms Interface - EBSCOhost Research Databases  Search Screen - Advanced Search  Database - PsycINFO 15,241  S22 DE "Running" Search modes - Find all my search terms Interface - EBSCOhost Research Databases  Search Screen - Advanced Search  Database - PsycINFO 2,312  S21 sport* Search modes - Find all my search terms Interface - EBSCOhost Research Databases  Search Screen - Advanced Search  Database - PsycINFO 51,447  S20 DE "Sports" Search modes - Find all my search terms Interface - EBSCOhost Research Databases  Search Screen - Advanced Search  Database - PsycINFO 17,497  S19 DE "Physical Endurance" Search modes - Find all my search terms Interface - EBSCOhost Research Databases  Search Screen - Advanced Search  Database - PsycINFO 1,160  S18 DE "Sports Medicine" OR DE "Physical Treatment Methods" OR DE "Physical Strength" OR DE "Physical Agility" OR DE "Physical Therapy" OR DE "Physical Health Assessment" Search modes - Find all my search terms Interface - EBSCOhost Research Databases  Search Screen - Advanced Search  Database - PsycINFO 6,618  S17 walk* Search modes - Find all my search terms Interface - EBSCOhost Research Databases  Search Screen - Advanced Search  Database - PsycINFO 35,164  S16 DE "Walking" Search modes - Find all my search terms Interface - EBSCOhost Research Databases  Search Screen - Advanced Search  Database - PsycINFO 6,830  S15 endurance Search modes - Find all my search terms Interface - EBSCOhost Research Databases  Search Screen - Advanced Search  Database - PsycINFO 4,856  S14 rehabilitat* Search modes - Find all my search terms Interface - EBSCOhost Research Databases  Search Screen - Advanced Search  Database - PsycINFO 161,358  S13 graded N2 activit* Search modes - Find all my search terms Interface - EBSCOhost Research Databases  Search Screen - Advanced Search  Database - PsycINFO 134  S12 "physically fit" or "physically active" Search modes - Find all my search terms Interface - EBSCOhost Research Databases  Search Screen - Advanced Search  Database - PsycINFO 2,371  S11 (physical N2 (exertion or training or fitness or movement or activity or activities or endurance or condition*)) Search modes - Find all my search terms Interface - EBSCOhost Research Databases  Search Screen - Advanced Search  Database - PsycINFO 48,567  S10 "resistance training" or "strength training" Search modes - Find all my search terms Interface - EBSCOhost Research Databases  Search Screen - Advanced Search  Database - PsycINFO 1,134  S9 "weight bearing" or "weight lifting" Search modes - Find all my search terms Interface - EBSCOhost Research Databases  Search Screen - Advanced Search  Database - PsycINFO 1,426  S8 aerobics or isometric* or pylometric* Search modes - Find all my search terms Interface - EBSCOhost Research Databases  Search Screen - Advanced Search  Database - PsycINFO 6,268  S7 prehabilitat* or "pre-habilitat*" or "pre habilitat" Search modes - Find all my search terms Interface - EBSCOhost Research Databases  Search Screen - Advanced Search  Database - PsycINFO 18  S6 exercis* Search modes - Find all my search terms Interface - EBSCOhost Research Databases  Search Screen - Advanced Search  Database - PsycINFO 77,623  S5 (accelerometer* or accelerometry or pedometer*) Search modes - Find all my search terms Interface - EBSCOhost Research Databases  Search Screen - Advanced Search  Database - PsycINFO 3,148  S4 DE "Exercise" OR DE "Physical Activity" OR DE "Actigraphy" OR DE "Exercise" OR DE "Aerobic Exercise" OR DE "Weightlifting" OR DE "Movement Therapy" OR DE "Physical Fitness" Search modes - Find all my search terms Interface - EBSCOhost Research Databases  Search Screen - Advanced Search  Database - PsycINFO 39,896  S3 S1 OR S2 Search modes - Find all my search terms Interface - EBSCOhost Research Databases  Search Screen - Advanced Search  Database - PsycINFO 12,189  S2 ("cancer patient*") Limiters - Language: English; Document Type: Journal Article  Search modes - Boolean/Phrase Interface - EBSCOhost Research Databases  Search Screen - Advanced Search  Database - PsycINFO 9,193  S1 ("cancer surviv*") Limiters - Language: English; Document Type: Journal Article  Search modes - Boolean/Phrase Interface - EBSCOhost Research Databases  Search Screen - Advanced Search  Database - PsycINFO 3,594 | 2118 |
